# Lens-induced uveitis in a patient with hypermature cataract

**DOI:** 10.22336/rjo.2021.62

**Published:** 2021

**Authors:** Raluca Bievel-Rădulescu, Bogdana Tăbăcaru, Horia Tudor Stanca

**Affiliations:** * “Prof. Dr. Agrippa Ionescu” Clinical Emergency Hospital, Bucharest, Romania; ** “Carol Davila” University of Medicine and Pharmacy, Bucharest, Romania

**Keywords:** hypermature cataract, lens-induced uveitis, phacogenic uveitis, phacolytic glaucoma

## Abstract

**Objective:** Our paper aims to report an unusual case of phacogenic uveitis with secondary glaucoma occurring after spontaneous rupture of the lens capsule in a patient with hypermature age-related cataract, to describe its particularities and to review the classification of the lens-induced uveitis.

**Methods:** We described the case of an 83-year-old male with a history of hypermature cataract, who presented to our clinic for right eye pain. Examination revealed circumciliary congestion, diffuse corneal edema, lens debris floating in the anterior chamber, pseudohypopyon and the opacification of the whole lens. An elevated intraocular pressure was also associated. We did not observe any history of previous intraocular surgery or trauma.

**Results:** We diagnosed the case as a phacogenic uveitis with secondary glaucoma and we planned to remove the inflammation trigger, namely the lens and its fragments by the most adequate technique, in order to control the inflammation and the IOP spikes, to alleviate the pain and to improve the patient’s visual function and the quality of life.

**Conclusions:** Phacogenic uveitis may present a cloudy cornea and a turbid anterior chamber that mimics endophthalmitis. Careful examination, medical history and ancillary investigations are helpful in establishing an accurate diagnosis and the appropriate treatment can reduce or eliminate the inflammation completely, decrease the intraocular pressure, being able to allow, depending on the particularities of the case, the visual gain.

## Introduction

Currently, cataract surgery is performed earlier than before due to the progress of technology and better outreach programs, so complications of hypermature cataracts are rare. However, we still meet Morgagnian cataract and its complications in some parts of the developing countries of the world. The lens can become a source of intraocular inflammation through surgical or traumatic rupture of the capsule or by leakage of lens proteins through an intact capsule.

This case report described a case of phacoanaphylactic uveitis presenting as diffuse corneal haze and raised intraocular pressure with turbid fluid in the anterior chamber due to a spontaneous rupture in the anterior lens capsule. 

## Case report

**Fig. 1:**
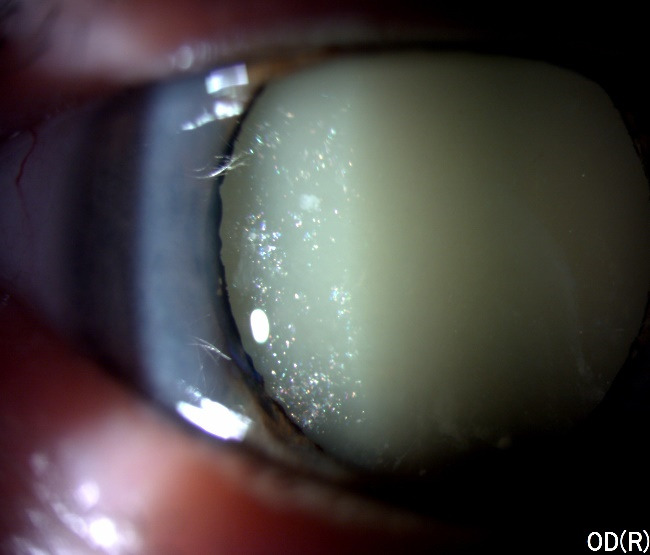
Slit-lamp examination of the right eye

**Fig. 2:**
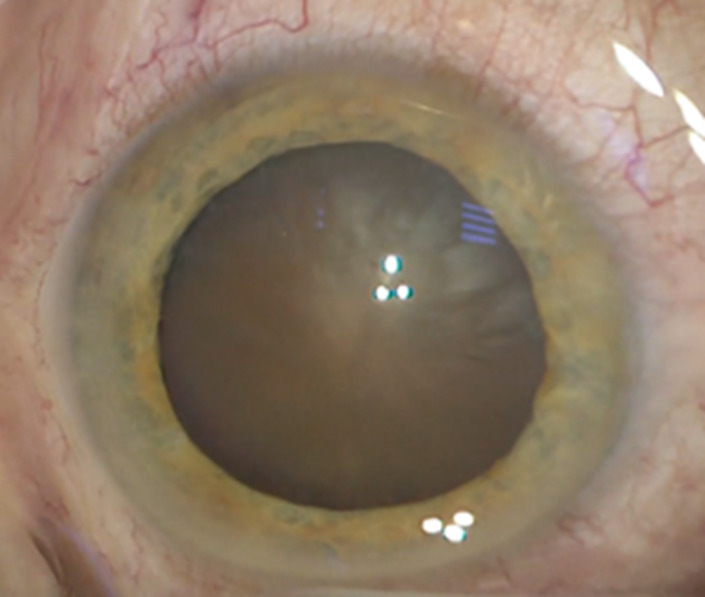
Intraoperative aspect of the left eye

An 83-year-old Caucasian male, farmer, presented to our Department of Ophthalmology with complaints of sudden onset pain and redness in his right eye. The patient did not offer any history of ocular trauma or previous ocular surgery. He had no relevant family history or ophthalmological afflictions. He was not taking any topical or systemic medication. The patient had been known to our clinic for 3 months when he was diagnosed with hypermature cataract in the right eye (**Fig. 1**) and mature cataract in the left eye (**Fig. 2**). He was supposed to undergo a cataract extraction in the left eye first, and then in the right eye, according to his demands.

The patient came back for the planned intervention of cataract surgery in the left eye, but during the preoperative consultation he complained about acute ocular pain in the right eye and photophobia with an abrupt onset just a few days before.

**Fig. 3:**
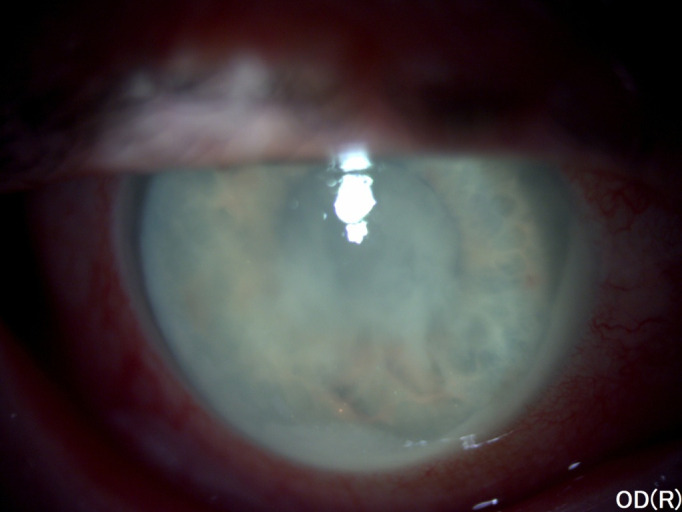
Right eye anterior segment aspect at presentation

**Fig. 4:**
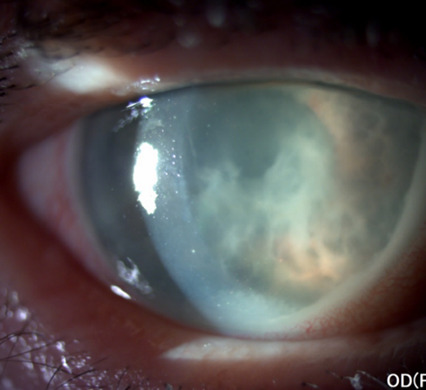
Slit-lamp examination of the right eye at presentation

At the clinical examination, visual acuity was light perception in the right eye and counting fingers in the left one. Intraocular pressure was 50 mmHg GAT in the right eye and 13 mmHg GAT in the left eye. Slit lamp biomicroscopy in the right eye revealed circumciliary congestion, diffuse corneal edema, pseudohypopyon, the opacification of the whole lens (**Fig. 3**) with the presence of suspended white particles in the anterior chamber and in the pupillary area (**Fig. 4**).

We further proceed with other investigations in order to establish a diagnosis and the course of treatment.

**Fig. 5:**
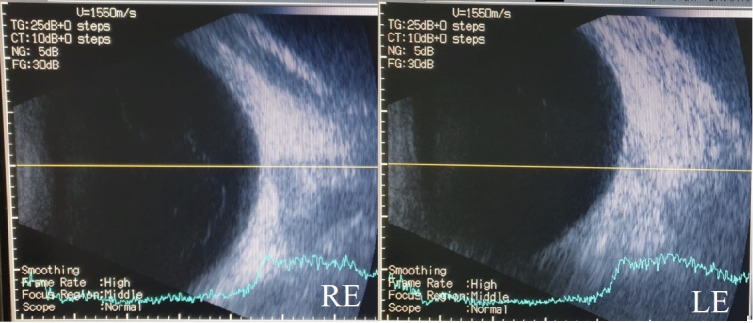
Ocular Ultrasonography (RE-Right eye; LE- Left eye)

The ultrasonography for both eyes revealed no pathological changes except for a hyper-reflective band between the middle and posterior third of the vitreous in the right eye, probably due to the detached posterior hyaloid (**Fig. 5**).

Based on the anamnesis, the eye exam, and the ultrasound aspect of the globe, the first stage diagnosis was lens induced-uveitis.

The differential diagnosis included causes of other lens-induced glaucoma, as well as other causes of hypopyon. Acute infectious endophthalmitis, toxic anterior segment syndrome (TASS), and flare-up of pre-existing uveitis were also taken into consideration (**Table 1**). 

**Tabel 1:** Differential diagnosis of lens-associated anterior-segment inflammation, related to the presented-case

Differential Diagnosis	Common with the case	Exclusion factors
Phacomorphic Glaucoma	- Elevated intraocular pressure;	- Normal depth of the anterior chamber
	- Edematous cornea.	
Lens-Particle Glaucoma	- Conjunctival injection;	- No history of recent intraocular surgery or trauma
	- Corneal edema;	
	- Elevated intraocular pressure.	
Behcet’s disease	- Redness;	- No oral aphthous ulcers;
	- Photophobia;	- No skin lesions;
	- Blurry vision.	- No genital ulcers.
Drug induced uveitis	- Pain;	- No utilization of:
	- Photophobia;	• Rifabutin;
	- Blurred vision;	• Cidofovir;
	- Redness.	• Bisphosphonates;
		• Sulfonamides;
		• Topical agents (Metipranolol, Brimonidine).
Acute infectious endophthalmitis	- Decreased visual acuity;	- Patient denies eye trauma or surgery
	- Photophobia;	
	- Conjunctival;	
	- Hyperemia.	
Toxic anterior segment syndrome (TASS)	- Decreased visual acuity;	- No history of intraocular surgery
	- Diffuse corneal edema;	
	- Photophobia;	
	- Elevated intraocular pressure.	
Flare-up of pre-existing uveitis	- Decreased visual acuity;	- No history of uveitis, no systemic or ocular signs of uveitis
	- Prominent cell and flare reaction.	

**Fig. 6:**
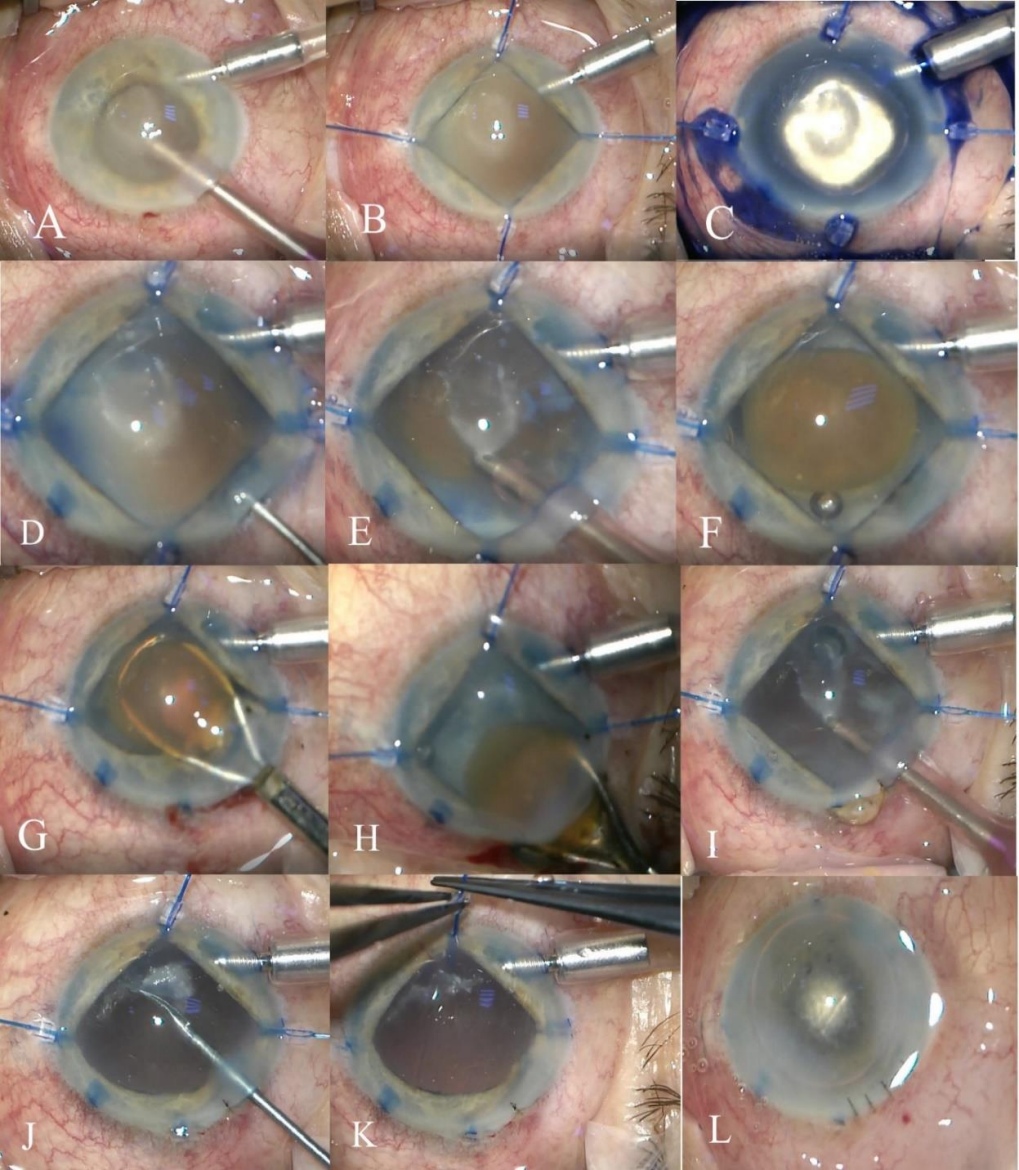
Intraoperative steps: **A** - aspiration of the lenticular matter from the anterior chamber; **B** - mechanical pupil dilation using iris-hooks; **C** - staining the anterior capsule by Trypan Blue dye; **D** - evidence of the area of the spontaneously ruptured anterior capsule; **E** - cortex aspiration; **F** -hydrodissection; **G, H** - manual extracapsular extraction of the opaque lens; **I** - Irrigation and aspiration of residual cortical lens matter; **J** - removal of the fibrotic material with a 23-gauge curved scissor; **K** - removal of the iris hooks; **L** - reformation of the anterior chamber by air

The goal of immediate treatment was to reduce inflammation and the IOP spikes, for which systemic ocular hypotensive therapy (intravenous mannitol and oral acetazolamide) was administrated. We also initiated the combined regimen of topical steroids and topical antiglaucoma drugs (prostaglandin-analog + beta-blocker + carbonic anhydrase inhibitor). The cataract extraction was delayed for 2 days until moderate control of inflammation and intraocular pressure allowed the procedure to be safe. The patient subsequently underwent manual extracapsular cataract extraction. The surgery was performed under local anesthesia with peribulbar block and consisted of: aspiration of the lenticular matter from the anterior chamber, mechanical pupil dilation using iris-hooks, staining the anterior capsule by Trypan Blue dye, evidence of the area of the spontaneously ruptured anterior capsule, aspiration of the cortical matter, gentle hydrodissection and manual extracapsular extraction of the opaque lens, irrigation and aspiration of the residual cortical lens matter, removal of the fibrotic material with a 23-gauge curved scissor, removal of the iris hooks, corneal suturing and reformation of the anterior chamber by BSS and sterile air (**Fig. 6**). 

Implantation of an intraocular lens was not performed as the patient did not consent prior to surgery. No intraoperative complication was encountered.

**Fig. 7:**
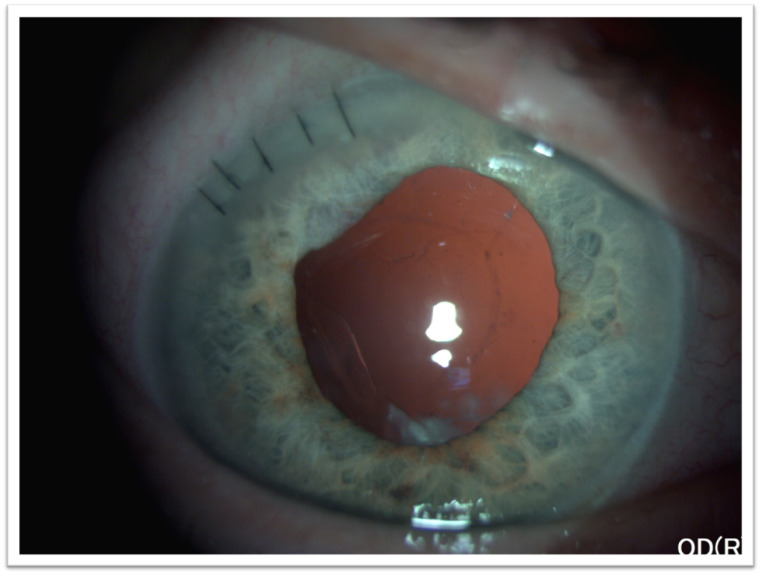
Anterior segment aspect of the right eye - postoperative at 1 month

Over the next month, the anterior chamber inflammation gradually resolved and the patient achieved a best-corrected visual acuity of 20/ 40 (Spherical equivalent of +12 Dsph) and an IOP of 17 mmHg (**Fig. 7**).

It is worth mentioning that the patient was very satisfied after the right eye cataract surgery because his vision improved significantly, even with the aphakia status and he did not consent for a secondary implantation of an IOL. 

Two months after, the patient returned to our clinic and requested the cataract surgery for the left eye and we performed phacoemulsification and PC IOL implantation. His visual prognosis was good with uncorrected visual acuity of 20/ 20 in the left eye.

## Discussion

Cataracts continue to be the leading cause of blindness in many countries [**1**-**3**]. Even if the cost-effectiveness of the procedure is high and the technique is standardized for most cases, the addressability of patients for surgery remains an important issue in some countries. This has led to many patients with mature and hypermature cataract. Untreated cataract can progress to hypermature cataract with accelerated dissolution of lens fibers, liquefaction of the entire cortex, and a small hard nucleus covered by a fragile capsule [**1**-**3**]. In case of a hypermature or Morgagnian cataract, the entire cortex is liquefied and the lens is transformed into a mass of milky fluid. The small nucleus usually settles at the bottom. Hypermature cataracts can lead to several complications, including: phacolytic glaucoma, phacogenic uveitis and dislocation of the lens or nucleus [**2**,**3**].

Lens-induced uveitis (LIU) represents a relatively rare disease (< 1% of all cases of uveitis) and can occur after spontaneous rupture of the capsule in case of hypermature age-related cataract, or in other situations such as rupture of the anterior lens capsule following trauma [**4**-**7**].

Nowadays, LIU can be classified based on the integrity of the capsule. When the capsule is ruptured, the terms phacogenic or phacoanaphylactic uveitis are used [**6**-**8**]. The term “phacolytic” uveitis or “phacolytic” glaucoma is used when the capsule is macroscopically intact, but there is a leakage of lens proteins that causes a non-granulomatous inflammatory reaction [**7**,**10**].

Phacotoxic uveitis or phacogenic non-granulomatous uveitis represents a chronic intraocular inflammation related to lens proteins after rupture of the lens capsule [**10**].

Lens-induced glaucoma is a secondary glaucoma in which the lens is involved in the mechanism of increased intraocular pressure. The glaucoma can occur in open-angle or angle-closure forms.

Lens induced glaucoma can be subdivided into two major categories. In the first category, the anterior flow of aqueous humor is blocked due to the lens. This category includes phacomorphic glaucoma (pupillary block glaucoma caused by an intumescent cataractous lens) and ectopia lentis (lens dislocation). The second category is characterized by the blocking of the trabecular network by lens proteins (phacolytic glaucoma), lens material or debris or by phacoanaphylactic response to the lens material [**11**-**13**].

Pupillary block glaucoma is characterized by the obstruction of the aqueous outflow by the apposition of the iris root to the trabecular meshwork. A senile cataractous lens that has progressed enough to become intumescent, has an increased anteroposterior length, and can cause pupillary block. The iridolenticular apposition interrupts the normal flow of aqueous humor from the anterior chamber to the posterior chamber. This leads to an accumulation of aqueous humor in the posterior chamber, pushing the iris root forward, eventually leading to an angle closure [**14**].

Phacoanaphylactic glaucoma occurs in the setting of a ruptured lens capsule and is characterized by a granulomatous antigenic reaction to lens protein that leaks from the capsule after trauma or is retained as lens fragments after intraocular surgery [**11**].

Usually, the etiological diagnosis is quite difficult. Though neovascular glaucoma can be seen in patients with diabetes, this patient had no neovascularization of the iris or angle to suggest this diagnosis. Aqueous misdirection typically occurs in the post-operative setting. Phacolytic glaucoma is a form of open-angle glaucoma that is caused by the leakage of the lens material from an intact, though perhaps degenerate, capsule of a mature or hypermature cataract.

The eye is considered a privileged immune organ. The lens is isolated from the fetal circulation early in the embryonic life, being devoid of vascularization and innervation. Nowadays, lens-induced uveitis is recognized as an autoimmune disease resulting from an abnormal recognition of lens proteins as non-self. The immunopathogenesis of lens-induced uveitis is the result of self-sensitization to the lens proteins. After rupture of the capsule and sensitization of the lens proteins, an inflammatory reaction is formed either granulomatous or non-granulomatous and involves a hypersensitivity reaction (type II, type III, or T-cell mediated) [**6**-**9**,**19**].

In most of the cases, LIU remains a challenging diagnosis. In order to obtain the right diagnosis, the clinicians should perform needle biopsy and histopathology. Aqueous humor cytology can be helpful in elucidating the correct diagnosis. Cytopathological examination may reveal a zonal granulomatous inflammatory reaction, with polymorphonuclear leukocytes surrounding a nidus of damaged or retained material lens [**18**].

An important part of the diagnosis is to determine the status of the lens capsule, whether it is intact or disrupted [**5**,**8**,**20**]. When it is available, scanning electron microscopy can detect a possible rupture of the anterior lens capsule [**14**-**18**].

In our case, the patient with hypermature cataract in the right eye had a spontaneous rupture of the anterior capsule with the release of the lenticular particles in the anterior chamber, which led to an immune reaction. 

Lens-induced uveitis represents an emergency condition that needs cataract extraction without delay. It is imperative to control intraocular pressure with medical therapy before surgery to prevent possible complications such as suprachoroidal effusions or hemorrhage and expulsive hemorrhage due to sudden decompression of the globe [**20**-**23**]. In order to control the inflammation and the IOP spikes, intra- or extra-capsular cataract extraction must be performed. Among the intraoperative complications that may occur in these patients are: posterior capsular rent, zonular dehiscence, vitreous loss, etc. Phacoemulsification can be difficult to perform, given the lax zonules and the hard lens due to the hypermature cataract. Under these conditions, a better choice may be manual ECCE or a small incision cataract surgery [**21**-**23**]. Irvine was the first to support the idea of extracapsular surgery in 1957, affirming that this technique could prevent forward movement of the vitreous and therefore loss of the vitreous [**8**-**10**]. In a study performed in India, Singh et al. have shown that ECCE with PC IOL implantation is a safe and effective method of visual rehabilitation in cases of LIU. With a follow-up period of two years, all patients maintained a normal postoperative intraocular pressure of less than 20 mmHg without any additional medical therapy [**22**]. The efficacy of manual small incision cataract surgery was demonstrated in a study conducted by Venkatesh et al. on 33 patients who showed that this may be a safe and effective method of cataract extraction for patients with phacolytic glaucoma [**23**].

Cataract surgery is the definitive treatment of phacogenic uveitis. Trabeculectomy may lower the intraocular pressure but it does not remove the inciting agent of the increased intraocular pressure, namely the cataract itself. 

There is increased risk of expulsive hemorrhage and/ or choroidal effusion with elevated perioperative intraocular pressures. Therefore, lowering the IOP to physiologic range is indicated pre-operatively with topical and oral agents.

**Conflict of Interest statement**

The authors state no conflict of interest.

**Informed Consent and Human and Animal Rights statement**

Informed consent has been obtained from all individuals included in this study.

**Authorization for the use of human subjects**

Ethical approval: The research related to human use complies with all the relevant national regulations, institutional policies, is in accordance with the tenets of the Helsinki Declaration, and has been approved by the review board of “Prof. Dr. Agrippa Ionescu” Clinical Emergency Hospital, Bucharest, Romania.

**Acknowledgements**

Not applicable.

**Sources of Funding**

None of the authors has any financial or proprietary interests to disclose.

**Disclosures**

None.
